# Research on the Generation of High-Purity Vortex Beams Aided by Genetic Algorithms

**DOI:** 10.3390/nano15181448

**Published:** 2025-09-19

**Authors:** Xinyu Ma, Wenjie Guo, Qing’an Sun, Xuesong Deng, Hang Yu, Lixia Yang

**Affiliations:** Information Materials and Intelligent Sensing Laboratory of Anhui Province, School of Electronic and Information Engineering, Anhui University, Hefei 230601, China; p23301199@stu.ahu.edu.cn (X.M.); p23301201@stu.ahu.edu.cn (W.G.); dxs_527@163.com (X.D.); yuhang232425@163.com (H.Y.);

**Keywords:** vortex beams, spin–orbit interaction, geometric phase

## Abstract

Vortex beams (VBs) generated by plasmonic metasurfaces hold great potential in the field of information transmission due to their unique helical phase wavefronts and infinite eigenstates. However, achieving perfect multiplexing and superposition of VBs with different orders remains a challenging issue in nanophotonics research. In this paper, based on a single-layer metallic porous metasurface structure applicable to the infrared spectrum, VBs with orders 2, 4, 6, and 8 are realized through the arrangement of annular elliptical apertures. Moreover, perfect VBs are achieved by optimizing key structural parameters using a genetic algorithm. The optimization of key structural parameters via genetic-based optimization algorithms to attain the desired effects can significantly reduce the workload of manual parameter adjustment. In addition, leveraging the orthogonality between VBs of different orders, concentric circular multi-channel VBs array (*l* = 2, 6) and (*l* = 4, 8) are realized. High-purity multiplexing architectures (>90%) are achieved via rational optimization of critical structural parameters using a genetic optimization algorithm, which further mitigates information crosstalk in optical communication transmission. The introduction of the genetic algorithm not only reduces the workload of manual arrangement of unit arrays but also enables the generation of more perfect VBs, providing a new research direction for optical communication transmission and optical communication encryption.

## 1. Introduction

Orbital angular momentum (OAM)-carrying photons, as revolutionary information carriers in optical communication and quantum technology, have broken through the technical framework of traditional amplitude, frequency, and polarization encoding, providing a new degree of freedom for information transmission [1,2]. Beyond their applications in information fields, they also exhibit remarkable potential in optical micromanipulation—a capability rooted in their unique mechanical properties. Vortex beams (VBs)—the physical form of such photons—feature unique helical phase wavefronts ($e^{il\varphi }$, where *l* denotes the unbounded topological charge with arbitrary values and *φ* is the azimuthal angle); theoretically, they possess infinite orthogonal eigenstates, laying the foundation for high-dimensional information encoding. Their typical ‘doughnut-shaped’ annular intensity distribution not only enables selectivity in spatial manipulation for information processing but also supports precise capture, rotation, and transportation of microscale particles (e.g., dielectric spheres and biological cells) in optical micromanipulation scenarios. Meanwhile, the inherent orthogonality between different topological charge orders supports multi-channel multiplexing [3,4]; critically, the quantized nature of OAM (each photon carries *l*ℏ) endows it with prominent advantages in improving channel capacity and encryption security—enabling a leap in communication rates via parallel transmission of different orders and facilitating the construction of highly secure quantum encryption systems relying on mode orthogonality [4]—while in optical micromanipulation, this quantized angular momentum allows for controllable angular momentum transfer to target micro-objects, realizing accurate rotational manipulation with tunable speed and direction [5]. These integrated properties make OAM-carrying photons of landmark significance for next-generation optical communication networks, quantum information systems, and advanced optical micromanipulation technologies.

Metasurfaces, as two-dimensional electromagnetic materials composed of subwavelength nanostructures, have revolutionized wavefront control by precisely regulating the phase, amplitude, and polarization of electromagnetic waves [6]; their inherent advantages—including broad spectral applicability (spanning the visible light to millimeter-wave bands) [7,8], ultrathin profiles (with thickness generally less than λ/5, where λ denotes the working wavelength), and favorable mechanical flexibility [9,10,11]—not only provide an ideal platform for the integrated generation and modulation of vortex beams (VBs), but also effectively compensate for the performance shortcomings of traditional VB-generating/modulating devices in integrated optical scenarios. The core value of metasurfaces in this context lies in their ability to support the engineering application of VB-specific characteristics, thereby promoting the practical development of high-dimensional optical information systems. Notably, mode purity, as a critical performance metric for VBs, imposes scenario-specific stringent requirements: in long-haul optical communication systems (e.g., metropolitan area networks (MANs) and transoceanic optical fiber communications), the mode purity of VBs typically needs to exceed 92% to mitigate signal attenuation and modal dispersion [12]; in quantum information processing (e.g., quantum key distribution (QKD) and VB-based quantum state transmission), it requires a higher threshold of 95% or above to maintain the fidelity of quantum states, given the extreme sensitivity of quantum states to environmental noise and inter-mode aliasing [13]; while in high-precision sensing applications, it generally needs to surpass 93%, as insufficient mode purity would introduce ‘background noise’ into detection signals, reducing the signal-to-noise ratio (SNR) and thus failing to meet the designed sensing accuracy [14]. Against this backdrop, the ongoing research on ultrasensitive vector displacement measurement conducted by Professor Luo Xiangang’s team necessitates the employment of a stable laser source, alongside the use of VBs with low noise and high mode purity to ensure measurement performance [15].

Based on the fundamental principle of photon spin–orbit interaction, this paper realizes the generation of high-purity VBs by annularly arranging elliptical apertures on a single-layer metallic metasurface. It is found that the structural parameters of the elliptical apertures and the annular arrangement parameters directly affect the generation of perfect VBs. Given the complexity of manual parameter adjustment and the limitations of errors easily introduced by manual calculations, this paper proposes a perfect VBs generation scheme based on an optimization algorithm. In this scheme, the major axis, minor axis, annular arrangement radius, and initial orientation angle of the elliptical apertures are taken as key optimization factors. Through a genetic-based optimization algorithm, the optimal ratio of each parameter is accurately calculated, successfully achieving the efficient generation of second-order, fourth-order, sixth-order, and eighth-order VBs. The effectiveness of the scheme is verified by comparing the mode purity analysis with the simulation results.

Furthermore, to meet the demand for generating multi-channel VBs, this paper optimizes the parameters of concentric ring structures (*l* = 2, 6 and *l* = 4, 8) using the optimization algorithm, ultimately obtaining a multi-channel multiplexing scheme with high mode purity. Compared with traditional manual parameter adjustment (which relies on empirical trial-and-error and struggles with global optimization), genetic algorithms, by simulating the selection, crossover, and mutation mechanisms in biological evolution, exhibit advantages of strong global optimization capability and high robustness. They are particularly suitable for optimization scenarios involving multi-parameter coupling and nonlinear objective functions. Therefore, this paper selects the genetic algorithm to optimize the key structural parameters of the metasurface iteratively, enabling efficient acquisition of parameter combinations for high-purity VBs. The research results show broad application prospects in multiple fields such as optical communication encoding, optical integrated circuits, optical information multiplexing, and optical manipulation.

## 2. Theoretical Analysis

### 2.1. Geometric Phase

The geometric phase is an important basis for photon spin–orbit interaction. In this paper, the high-order Poincaré sphere is mainly used to describe and decompose arbitrary VBs [16], and the Pancharatnam–Berry (PB) phase in the Stokes parameter space is employed to characterize the spin-intrinsic OAM interaction.

A change in the polarization state of light introduces an additional phase. The normalized expression of incident circularly polarized light can be written as follows (taking left-handed circularly polarized light as an example):

(1) EinLCP=ExinEyin=cosφ+icos(φ+π2)=e−iφ=cosφ−sinφ=121−iσ,(σ=−1),

where σ=−1 denotes left-handed circular polarization (LCP) and σ=+1 denotes right-handed circular polarization (RCP). The generation of VBs via the PB phase can be explained using the Jones matrix [17]. Since circularly polarized light is an eigenstate of the spin angular momentum operator, the Jones transformation J(θ)=R(−θ)JR(θ) needs to be applied, where J is the Jones matrix, which can be rendered as $J=\left[\begin{array}{cc} J_{xx} & J_{xy}\\ J_{yx} & J_{yy} \end{array}\right]$, and the rotation transformation is denoted by the rotation matrix $R(\theta )=\left[\begin{array}{cc} cos\theta & -sin\theta \\ sin\theta & cos\theta \end{array}\right]$.

For normally incident LCP light, the Jones matrix is $J=\left[\begin{array}{cc} J_{xx} & 0\\ 0 & J_{yy} \end{array}\right]$*,* $J_{xy}=J_{yx}=0$. Therefore, the outgoing light after incident LCP light passes through a metal nanostructure with an orientation angle of can be described as follows:

(2) EoutLCP=ExoutEyout=Jθ21−iσ=122(Jxx+Jyy)1iσ+(Jxx−Jyy)e2iσθ1−iσ, σ=−1,

It can be seen from Equation (2) that the metasurface composed of multiple anisotropic structural units can make the outgoing light acquire an additional geometric phase of 2. Thus, it can be concluded that the change in geometric phase is twice the change in the orientation angle of the nanostructure [18].

### 2.2. Design of Genetic Algorithm Optimization Model

Genetic algorithm (GA) is a global optimization method rooted in natural selection and biological evolution, conducting iterative searches through simulating heredity, mutation, and selection processes. Its key advantage of handling discontinuous, non-differentiable objective functions [19] makes it suitable for high-dimensional, strongly coupled problems like metasurface parameter optimization [20]. While GA has been widely applied in beam shaping and metasurfaces, this work innovates in three aspects: (1) tailored modeling for concentric dual-order VBs with inter-order coupling; (2) GA parameter customization based on metasurface physical constraints; (3) achieving high purity for both high-order single and composite dual-order VBs. This section details the model design with emphasis on these contributions.

#### 2.2.1. Core Theoretical Elements and Parameter Selection Rationale

GA implementation requires mapping theoretical elements to optimization targets, with parameters determined by the literature and problem-specific characteristics. The key innovation lies in customizing GA parameters to address strong near-field coupling in metasurfaces, distinguishing it from generic applications:

Population and Individuals: Following standard GA encoding, each ‘individual’ represents a metasurface parameter set (chromosome) containing elliptical aperture dimensions (major/minor axes), orientation angle, and annular radius as ‘genes’.

Fitness Function: Uses VB mode purity as the core metric [21] with penalty terms for invalid aspect ratios, ensuring both purity and structural feasibility—addressing fabrication constraints often overlooked in mathematical optimization.

Genetic Operations: Uses roulette wheel selection [22] for high-fitness individuals, arithmetic crossover to balance exploration/exploitation, and, critically, 5% parameter perturbation to resolve strong parameter coupling and avoid premature convergence.

Convergence Mechanism: Stops when average fitness fluctuates <0.1% over 10 generations, balancing accuracy and efficiency based on 5–15 generation window tests.

**Figure 1:**
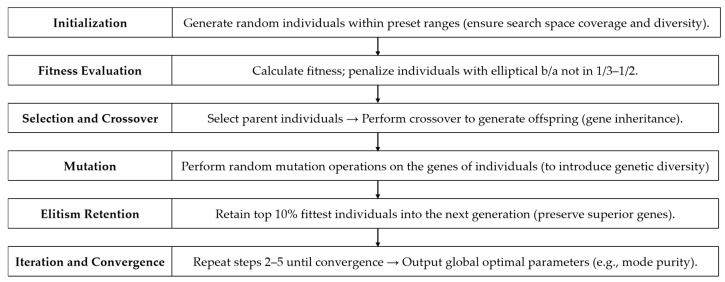
The 6-stage process of GA-based optimization model.

All models follow this logic, with implementation shown in Figure 1.

#### 2.2.2. Design of Single-Order Vortex Optimization Model

This single-objective model targets high-purity single-channel VBs (l=2, l=4, l=6 or l= 8), with a key innovation of >99% purity for high-order (l= 8) VBs—overcoming typical purity decay with topological charge:

Individual Encoding: Includes aperture dimensions (a,b), orientation angle (θ), annular radius (R), and gold layer thickness (h), initialized randomly for diversity.

Population Size: A total of 20 individuals/generation, achieving > 99% purity with moderate cost—optimized via 15/20/25 individual tests.

Fitness Function:

(3) $$fitness=\;penalty\;\times \;{purity}_{l}$$

where ${purity}_{l}$ enforces valid b/*a* ratios.

Optimization Process: Selection/crossover amplifies high-purity combinations, while mutation explores new spaces. For l= 8 VB, initial 60–80% purity evolves to >99% with mutation critical for escaping local optima—outperforming conventional methods with purity degradation.

Result Recording: Tracks per-generation fitness and purity, outputting parameters and VB phase/intensity distributions post-convergence.

#### 2.2.3. Design of Dual-Order Vortex Optimization Model

This multi-objective model optimizes concentric composite VBs (l=2, 6 or l=4, 8), addressing a gap in the literature focused on single-order optimization by handling inner-outer ring coupling:

Optimization Objectives: Maximizes both inner low-order (${purity}_{l1}$) and outer high-order (${purity}_{l2}$) purity—rarely explored in metasurface research.

Individual Encoding: A 7-variable encoding ($\theta_{1}$, $\theta_{2}$, $\theta_{3}$, $R_{1}$, $R_{2}$, a, b) captures coupling, with $\theta_{3}$ dedicated to eliminating composite phase defects.

Population and Iteration Settings: A total of 25 individuals over 120 generations—optimized via 20–30 individual and 100–150 generation tests to achieve > 95% dual purity with acceptable cost.

Fitness Function:

(4) $$fitness=\;penalty\;\times \;(w_{1}\times {purity}_{l1}+\;w_{2}\times \;{purity}_{l2})$$

with $w_{1}=w_{2}=0.5$ for balance, and a stricter penalty for both rings’ aspect ratios.

Optimization Effect: Crossover fuses optimal radii (avoiding phase mismatch) and mutation fine-tunes $\theta_{3}$ to eliminate defects—achieving >95% dual purity compared to conventional inter-mode interference issues.

Result Recording: Tracks dual purity convergence, validating composite VB quality via simulation.

#### 2.2.4. Parameter Sensitivity Analysis and Optimization Experiments

To verify the rationality of GA parameter settings, controlled univariate experiments were performed (fixing other parameters while varying the target one) [23]. For each parameter, a full value range was defined based on the traditional application boundaries of GAs in metasurface optimization and the physical constraints of the VB generation system [24]. Table 1 lists the optimal value for each parameter. Subsequent experimental results confirm that the GA parameter combination in Table 1 enables the algorithm to acquire the optimal parameter set for VBs.

#### 2.2.5. Summary of Optimization Models

This work advances GA-based optimization via three innovations: (1) tailored dual-order VB modeling with inter-ring coupling, addressing gaps in beam shaping; (2) physically informed parameters handling metasurface near-field interactions, distinct from generic frameworks; (3) >99% high-order single VB and >95% dual VB purity, overcoming typical high-order degradation. These establish a robust framework for high-quality vortex beam generation.

## 3. Design and Results

The rotation angle of nanostructures introduces a geometric phase, and the resulting phase change generates circular polarization that is either the same as or opposite to that of the incident light, which is reflected in the fact that the induced phase is twice the orientation angle. A certain number of metallic elliptical nanopores are arranged in a ring on the metasurface, and incident LCP/RCP light can generate VBs of different orders. Its construction equation is as follows:

(5) 2πl+Φ=2m×∆θ,

where θ is the rotation angle of the elliptical nanopore relative to the positive direction of the *y*-axis, and ∆θ is the difference in rotation angles between adjacent elliptical nanopores. Φ is added to offset the loss angle of the helical phase distribution of confined light caused by surface plasmon polaritons (SPPs) excited by different nanostructures, with $\Phi /k_{SPPs}=2\pi R-mP-m(a+b)/2$, where P is the simplified period of the nanopores. m is the number of pores around the center of the nanostructure, which can take values of 8, 12, 18, or 24.

**Figure 2:**
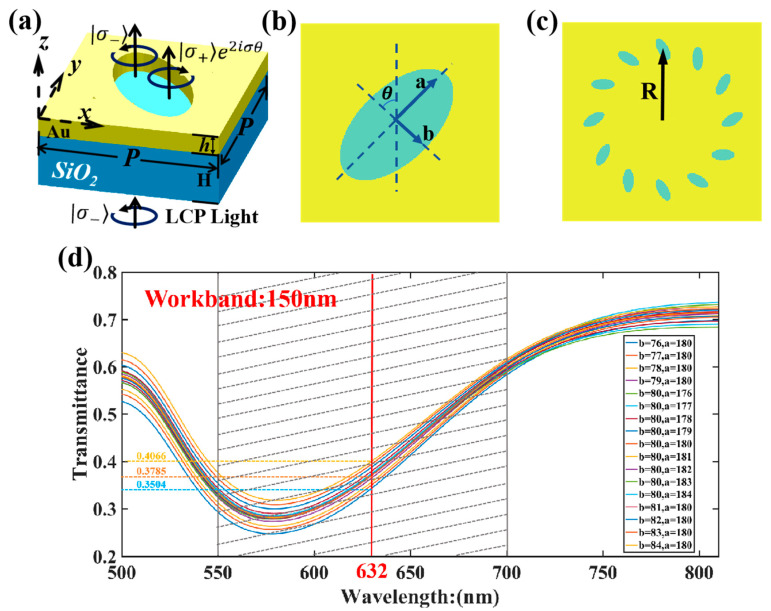
(**a**) Structure of the metasurface and elliptical pores. (**b**) Top view of elliptical aperture. (**c**) Top view of the second-order circular structure. (**d**) Transmittance diagrams of different long-axis and short-axis elliptical apertures.

In this study, a gold layer is deposited on a silica substrate, and elliptical apertures are etched in a ring arrangement on the gold layer. In the simulation design, the LCP light with a wavelength of 632 nm (red spectral band) is set at a distance of 500 nm from the gold layer, and the receiving plane is set at a distance of 500 nm from the gold layer. The structure of the metasurface and elliptical pores is shown in Figure 2, where a is the major axis of the elliptical pore, b is the minor axis of the elliptical pore, θ is the rotation angle of the elliptical nanopore relative to the positive direction of the *y*-axis, h is the thickness of the silicon dioxide (quartz) substrate (*h* = 2 μm), R is the radius of the ring arrangement, and m is the number of elliptical pores.

A physical environment similar to that in Lumerical and the Finite-Difference Time-Domain (FDTD) simulation algorithm is established in Matlab 2021b. In this study, the results of GA optimization in Matlab were substituted into Lumerical FDTD 2020 R2 software for simulation. It can be concluded that the vortex beam can have ultra-high mode purity after further optimization by the GA. The refractive index of the silica substrate is set to 1.5, and the dielectric constant of gold is determined using the Drude model [25]. From Figure 2a–c, the major axis a, minor axis b of the elliptical pore, gold layer thickness h, ring arrangement radius R, and initial rotation angle θ are taken as optimization variables. As illustrated in Figure 2d, within an error range of 4–5%, the transmittance of the elliptical aperture exhibits an extremely slight variation. This phenomenon demonstrates the excellent structural stability of the elliptical aperture. Furthermore, the operating wavelength range of the nanoaperture structure can cover 150 nm, manifesting prominent wide-band characteristics. The GA is used to optimize the aperture model, with the mode purity of the corresponding order as the optimization objective, to output parameter combinations with high mode purity (>90%).

Lumerical FDTD Solutions is a three-dimensional Maxwell equation-solving software, which is used to investigate the optical response of the proposed structure. Here, ‘simulation type’ is set to ‘3D’, ‘background index’ is set to ‘1’, ‘simulation time’ is set to ‘1000 fs’, ‘simulation accuracy’ is set to ‘4’, and ‘mesh step’ is set to ‘4 nm’. Next, add a rectangular glass substrate model, which is 4 × 4 × 2 ${\mu m}^{3}$ in size and has a refractive index of 1.5; add a rectangular gold film model with a size of 4 × 4 × 0.2 ${\mu m}^{3}$, and its dielectric constant comes from the study of Johnson and Christy. Add an elliptical nanohole in the center of the gold film, whose dielectric constant is set to ‘etch’. Then, copy and move nanoholes to form large rings, and adjust the orientation angle of each nanohole in turn according to the result calculated by Equation (5). Finally, add 632 nm circularly polarized incident light and monitors. Check that the absorption conditions of the perfectly matched layer are set at all boundaries, and the program can run.

Mode purity is determined by decomposing each OAM state using the Fourier transform. By matching circular collection points with phase shifts in integer multiples of two, the electric field components of the actual data in the x and y directions are collected. Finally, the energy proportion corresponding to the target order among all the possible states is obtained through integral calculation. The construction equations are as follows:

(6) $$A_{l}=\frac{1}{2\pi }\int_{0}^{2\pi }\psi (\varphi )e^{-il\varphi }d\varphi ,$$

(7) $$\psi (\varphi )=\sum_{l_{min}}^{l_{max}}(A_{l}e^{il\varphi }),$$

(8) $$P_{l}=\frac{A_{l}}{\sum_{l_{min}}^{l_{max}}A_{l}}$$

where φ denotes the azimuthal angle in polar or cylindrical coordinates, and ψ(φ) denotes the vector function sampled along the trajectory of the *z*-axis. $A_{l}$ represents the Fourier transform used to determine the specific order of the emergent light, and $P_{l}$ represents the total intensity-weighted beam energy. The normalized energy ratios are unitless, and their values indicate the proportion of each OAM mode [26].

### 3.1. Single-Channel VBs

**Figure 3:**
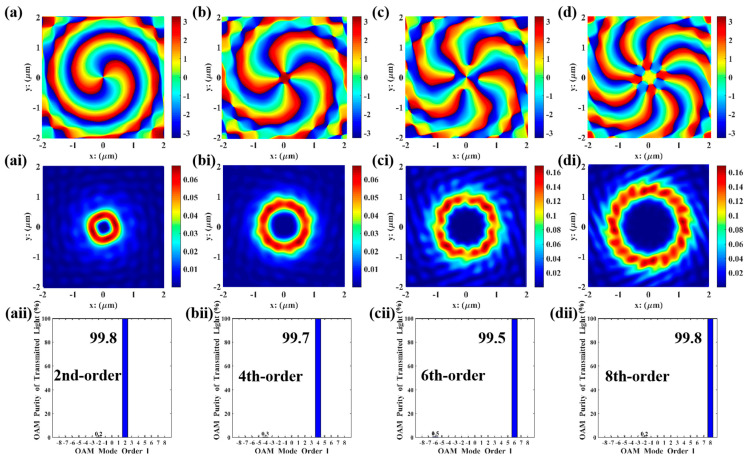
GA optimization of 2nd-, 4th-, 6th-, and 8th-order VBs: (**a**–**d**) the energy distribution of the focal ring of the transmitted light, measured in $V^{2}$/$m^{2}$; (**ai**–**di**) the electric field distribution of transmitted light in the y-direction, measured in V/m; (**aii**–**dii**) mode purity histograms.

Taking θ, a, b, h,and R as genetic factors, and setting the total mode purity of the specified order to be higher than 90% as the optimization objective, the above single-order vortex optimization model was adopted to perform parameter optimization for second-, fourth-, sixth-, and eighth-order vortices, respectively. During optimization, the parameter ranges for population initialization are major axis $a\;\epsilon \;\left[100,\;200\right]\;nm$, minor axis $b\;\epsilon \;\left[100,\;200\right]\;nm$, gold layer thickness $h\;\epsilon \;\left[100,200\right]nm$, annular radius $R\;\epsilon \;\left[500,\;1500\right]\;nm$, and initial orientation angle θ ϵ [0, 90°]. The parameter combinations of high-purity VBs for second, fourth, sixth, and eighth orders obtained via GA calculations are presented in Table 2. Meanwhile, Figure 3 shows the optimal result obtained by using the GA in Matlab. Each order of VBs exhibits a donut-shaped spiral phase wavefront, with a mode purity exceeding 90%.

**Figure 4:**
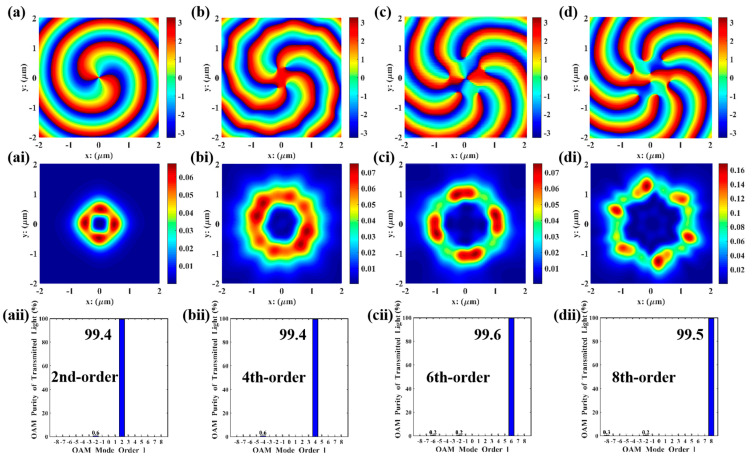
Lumerical FDTD simulations of 2nd−, 4th−, 6th−, and 8th−order VBs: (**a**−**d**) the energy distribution of the focal ring of the transmitted light, measured in $V^{2}$/$m^{2}$; (**ai**−**di**) the electric field distribution of transmitted light in the y-direction, measured in V/m; (**aii**−**dii**) mode purity histograms.

The FDTD simulations were performed in Lumerical using the parameter combinations in Table 2, and the results are shown in Figure 4. All orders of VBs were perfectly reproduced with high purity (>90%). Compared with the Matlab simulations in Figure 3, the phase distribution maps are smoother, and the vortex characteristics are more distinct. The electric field intensity distributions of the second, fourth, and sixth orders are similar to those in Matlab simulations, which is because the field distribution characteristics of low-order vortices are simpler and less sensitive to numerical errors and actual physical factors. However, in the 8th-order simulation, the beam propagates in ideal free space in Matlab simulations, where boundary limitations, material dispersion, absorption, or scattering are ignored; in contrast, in Lumerical simulations, actual boundary conditions as well as material dispersion and losses are considered, resulting in dissimilar electric field intensity distributions for the eighth order. In high-order mode, the memory requirement for FDTD simulation is approximately 12 GB. For Matlab, the required operating memory is mesh size-dependent; when the mesh size is rationally designed to match that of the FDTD simulation, the results of FDTD and Matlab are roughly consistent. Beyond this scenario, the MATLAB results are optimal solutions searched via the update and iteration of GA, leading to more idealized resultant graphs. In contrast, the results from Lumerical FDTD simulation software are derived from solving Maxwell’s equations, resulting in certain discrepancies between the two sets of results.

### 3.2. Multi-Channel VBs

Different orders of VBs exhibit high orthogonality, a property that enables the superposition of VBs of different orders. By aligning the near-field and far-field in the same propagation direction using different focal points, coupling phenomena of VBs with different orders can be achieved on homologous plasmonic metasurfaces. Higher-order VBs should have a larger focal radius on the same receiving plane. Our team has achieved three-channel VBs multiplexing and subsequent coding work [27]. In designing this scheme, mainly about two-channel, low-order VBs should be located in the inner ring, and high-order ones in the outer ring. Additionally, the number of nanopores and the annular radius are crucial to ensuring that two VBs of different orders can be superimposed with high orthogonality.

Multi-channel VBs can be expressed as a general superposition of multiple Laguerre-Gaussian (LG) modes with specific polarization states. The cylindrical coordinate expression of LG mode beams in free space can be written as Equation (9) [19]. Herein, the phase term $e^{\left[\frac{-ikr^{2}z}{2(z^{2}+z_{0}^{2})}\right]}$ contains the propagation-induced phase factor $e^{ikz}$, $w\left(z\right)=w_{0}\sqrt{1+{\left(\frac{z}{z_{0}}\right)}^{2}}$ Is the beam radius at propagation distance z, and $z_{0}=\pi w_{0}^{2}/\lambda$ is the Rayleigh length. The superposition of two VBs with different orders is shown in Equation (10), where $e^{i\delta_{l_{1}}}$ and $e^{i\delta_{l_{2}}}$ are the initial phases of the two VBs with different orders, respectively. Thus, the superposition equation of two VBs with different orders is expressed as follows:

(9) $$E_{LG}(r,\varphi ,z)=C_{lp}^{LG}\frac{w_{0}}{w(z)}(\frac{\sqrt{2}r}{w(z)}{)}^{\left|l\right|}e^{-\frac{r^{2}}{w^{2}(z)}}{\cdot e}^{\left[\frac{-ikr^{2}z}{2(z^{2}+z_{0}^{2})}\right]}\cdot e^{il\varphi },$$

(10) $${\left|L\left.G\right\rangle \right.}_{sup}=\frac{1}{\sqrt{c_{l_{1}}^{2}+c_{l_{2}}^{2}}}(c_{l_{1}}e^{i\delta_{l_{1}}}\left|L\left.G_{l_{1}}\right\rangle +c_{l_{2}}e^{i\delta_{l_{2}}}\left|L\left.G_{l_{2}}\right\rangle \right.\right.),$$

**Figure 5:**
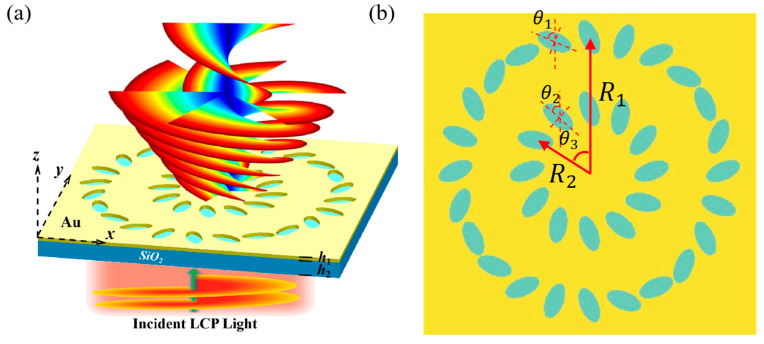
(**a**) Schematic diagram of multi-channel (*l* = 2, 6) VBs generation. (**b**) Schematic diagram of multi-channel concentric circular ring structures.

Based on the above principles, a physical environment and concentric circular ring structures were established in Matlab, with left circularly polarized light of 632 nm incident and received at the 500 nm plane. The propagation mode of the multi-channel (*l* = 2, 6) VBs is shown in Figure 5a. The concentric circular ring structure is shown in Figure 5b, where $\theta_{1}$ is the initial phase angle of the outer ring ellipses, $\theta_{2}$ is the initial phase angle of the inner ring ellipses, $\theta_{3}$ is the initial position angle difference between inner and outer ring ellipses, $R_{1}$ is the outer ring radius, $R_{2}$ is the inner ring radius, $m_{1}$ is the number of inner ring elliptical pores, and $m_{2}$ is the number of outer ring elliptical pores.

**Figure 6:**
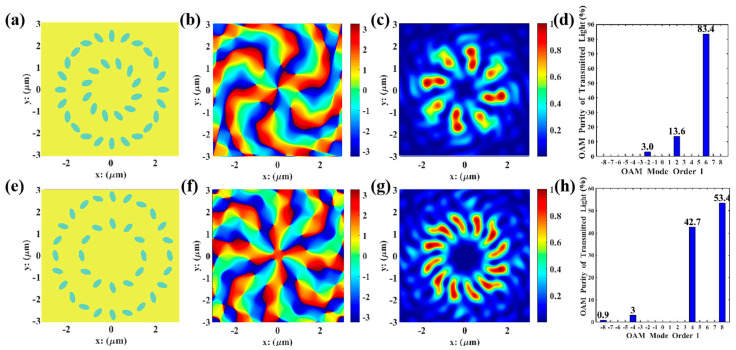
GA optimization of multi−channel l=2,6 and l=4,8 VBs: (**a**,**e**) elliptical pore distribution maps; (**b**,**f**) The energy distribution of the transmitted light, measured in $V^{2}$/$m^{2}$; (**c**,**g**) the electric field distribution of transmitted light in the y−direction, measured in V/m; (**d**,**h**) mode purity histograms.

Taking $\theta_{1}$, $\theta_{2}$, $\theta_{3}$, $R_{1}$, $R_{2}$, a, and *b* as genetic factors and setting the total mode purity of the specified orders to be higher than 90% as the optimization objective, the dual-order vortex optimization model was adopted to optimize parameters for the l=2, 6 and l=4, 8 multi-channel structures. As shown in Figure 6 below, during optimization, the fitness function is centered on the minimum value of dual-order purities to avoid one order having excessively high purity while the other being too low. The number of optimization variables increased to 7, and the parameter coupling was enhanced; thus, the number of iterations was increased to 120 generations, and the mutation probability was raised to 0.08 to expand the optimization range. The iteration results show that the l=2, 6 combination converged at the 90th generation, and the l=4, 8 combination at the 105th generation, with the final total mode purity of 97% and 96.1%, respectively. The optimized parameter combinations were obtained, and phase distribution maps, electric field distribution maps, and mode purity analyses indicate that both l=2, 6 and l=4, 8 multi-channel VBs have the final total mode purity exceeding 90% without phase defects, with the second and fourth orders in the inner rings clearly presented.

**Figure 7:**
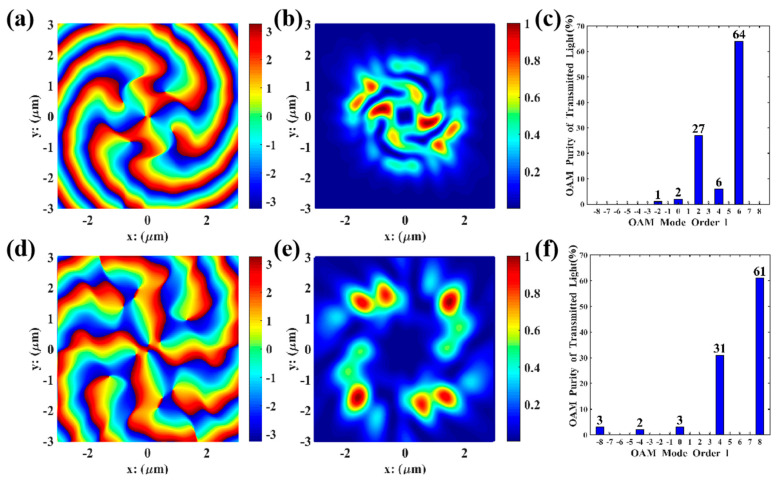
Lumerical simulations of multi−channel l=2,6 and l=4,8 VBs: (**a**,**d**) the energy distribution of the transmitted light, measured in $V^{2}$/$m^{2}$; (**b**,**e**) the electric field distribution of transmitted light in the y−direction, measured in V/m; (**c**,**f**) mode purity histograms.

As shown in Figure 6, both l=2, 6 and l=4, 8 multi-channel VBs have mode purities exceeding 90% without phase defects, with the second and fourth orders in the inner rings clearly presented. Therefore, the parameter combinations in Table 3 were substituted into Lumerical for FDTD simulations, as shown in Figure 7.

As shown in Figure 7, the l=2,6 and l=4,8 multi-channel VBs also have mode purities exceeding 90%. Compared with the Matlab simulations in Figure 6, the phase distribution maps from Lumerical are smoother, which is because Lumerical imposes stricter and more realistic constraints on boundary conditions, resulting in more reasonable electric field distributions. Additionally, it can be observed that the eighth-order electric field distribution in the l=4, 8 multi-channel VBs is an extension of the fourth-order one.

From Figure 6b,f and Figure 7a,d, the multi-channel VBs generated by this nanopore metasurface design exhibit minimal dispersion. Furthermore, the generated VBs feature high resolution and few phase defects; the second- and sixth-order VBs propagate in the same direction without any observed phase mismatch, with their focal ring energy remaining highly concentrated and well-defined boundaries. Similarly, the nanopores for the fourth- and eighth-order VBs have consistent orientations and significant symmetry, and the reduced focal ring radius leads to more concentrated transmitted energy without phase deviation. From Figure 6d,h and Figure 7c,f, the total purity of the target OAM modes is extremely high, all exceeding 90%. This indicates that in the composite VBs generated by each concentric circular structure, the target order VBs account for a significant proportion of the energy, leading to the conclusion that this design can generate high-quality, high-purity VBs. The core of forming multi-channel VBs lies in utilizing the reusability and high orthogonality between VBs of different orders, a property that can be further applied to the design of homologous co-directional composite VBs with higher orders and more channels.

## 4. Conclusions

Based on a single-layer metallic porous metasurface structure, this study successfully generated high-purity single-order and multi-channel VBs by combining GA to optimize key structural parameters. The research verified the efficiency of GA in handling multi-dimensional, strongly coupled parameter optimization, finding parameter combinations with high mode purity for second-, fourth-, sixth-, and eighth-order single-order VBs. Meanwhile, utilizing the orthogonality of VBs with different orders, the multiplexing of l=2, 6 and l=4, 8 multi-channel VBs was realized through a dual-order GA model, with the total mode purity exceeding 90%. A complete optimization process and simulation verification system were also established. This achievement provides a new path for high-dimensional information transmission and multiplexing in the field of optical communication, and has potential application value in areas such as optical communication coding and quantum encryption. In the future, it can be further extended to the optimization and experimental verification of higher-order VBs.

## Figures and Tables

**Table 1:** Parameter combinations of GA.

Parameter Category	Variable Name	Parameter Setting
Genetic operation parameters	Selection method	Roulette wheel selection
	Crossover method	Arithmetic crossover
	Crossover probability	0.7
	Mutation method	Random perturbation mutation
	Mutation probability	0.15
	Mutation perturbation range	5% of the parameter search range
Convergence mechanism parameters	Convergence condition	Population average fitness value fluctuates <0.1% over 5 consecutive gens
Fitness function parameters	Fitness core index	Purity of vb mode
	Fitness penalty coeff valid	1.0
	Fitness penalty coeff invalid	0.1
	Fitness penalty condition	Elliptical hole aspect ratio (b/a) not in 1/3–1/2

**Table 2:** Parameter combinations optimized by GA in single-channel VBs.

l	m	$a_{1}$ (nm)	$b_{1}$ (nm)	θ (°)	*h* (nm)	*R* (nm)	Purity (%)
2	12	160	80	30	160	660	99.8
4	12	160	80	25	160	745	99.7
6	24	160	80	15	160	1100	99.5
8	24	160	80	25	160	1200	99.8

**Table 3:** Parameter combinations optimized by GA in multi-channel VBs.

l	*m*_1_	*m*_2_	$a_{1}$ (nm)	$b_{1}$ (nm)	$\theta_{1}$ (°)	$\theta_{2}$ (°)	$\theta_{3}$ (°)	*R*_1_ (nm)	*R*_2_ (nm)	Purity (%)
2, 6	12	24	160	80	25	5	15	1180	560	97.0
4, 8	12	24	160	80	15	15	0	1510	800	96.1

## Data Availability

The data that support the findings of this study are available from the corresponding author upon reasonable request.
